# Correction: A theoretical framework to understand high electron mobilities in cable bacteria

**DOI:** 10.1039/d6sc90073a

**Published:** 2026-03-31

**Authors:** Andrew J. Smith, David N. Beratan

**Affiliations:** a Department of Chemistry, Duke University Durham NC 27708 USA; b Department of Physics, Duke University Durham NC 27708 USA; c Department of Biochemistry, Duke University Durham NC 27710 USA andrew.smith@duke.edu david.beratan@duke.edu

## Abstract

Correction for ‘A theoretical framework to understand high electron mobilities in cable bacteria' by Andrew J. Smith *et al.*, *Chem. Sci.*, 2026, **17**, 5442–5450, https://doi.org/10.1039/D5SC04393J.

The authors recently discovered some errors in this publication that warrant correction and additional discussion, as detailed below.

## 1 Corrections

### 1.1 Main text

In Section 2.2 of the original paper, where the time convention *m*/*k* is discussed, the following statement should be added (referring to cable bacteria as CB):

 “Although the convention of measuring elapsed time yields accurate results for CB (see correction Section 2), it is not valid for all systems.”

The sentence:

 “One-dimensional approximations to the hopping transport in arms of the CB may overestimate the mobility, because mobility drops with transport network dimensionality in periodic structures.”

should be replaced by:

 “One-dimensional approximations to the hopping transport in CB may overestimate the mobility, since mobility decreases in the presence of obstacles (*i.e.*, points where the particle is not free to move in the direction of interest).”

Effects attributed to dimensionality arise rather from the step-counting time convention when diffusion is projected onto a fixed direction. The following addition should be made at the end of Section 2.3:

 “Using the definition of elapsed time as *m*/*k* suppresses errors that arise from coarse-graining (see correction Section 2).”

### 1.2 Supplementary information (SI)

#### 1.2.1 SI Section S4A

The corrected version of Fig. S4, based on the original step counting convention, is shown below (points that did not reach convergence are omitted). The interpretation of Fig. S4 is unchanged.


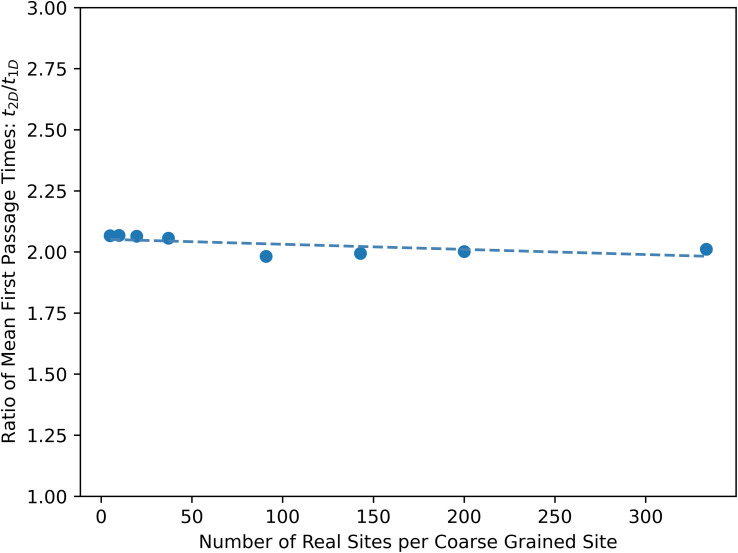
 **Fig. S4-R** When counting time steps, the expected ratio of MFPTs for a 2D grid and 1D chain is 2. Consistent with this expectation, all of the converged calculations of the ratio of 2D grid and 1D chain MFPTs are within the convergence threshold (5%) of two.

#### 1.2.2 SI Section S5

In the original manuscript, we computed the ratio of MFPTs for a 3D model of a CB conduction channel and compared it with that of a 1D chain. We found an MFPT ratio of 2.61 between the 3D conduction-channel model and the 1D chain model. Because this comparison involved a single conduction channel without obstacles, recomputation of the MFPT ratio using the mean escape time convention yields a ratio of unity. Thus, if the three-dimensional structure of CB conduction channels resembles the model considered here (*i.e.*, contains no obstacles along the transport direction), treating the conduction pathway as a more complex 3D channel rather than as a 1D chain does not change the results presented in the main text, and Fig. S7 is identical to the data published in Fig. 6.

The text:

 “Performing an unbiased random walk on the short 3D fiber model and the short 1D fiber model produces a ratio of MFPTs 
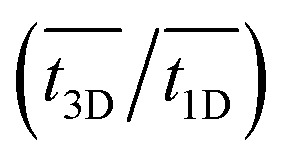
 of 2.61. Applying the scaling ratio of MFPTs leads to a nearly identical range of possible carrier hopping parameter values (as shown in Fig. S7).”

should read:

 “Performing an unbiased random walk with a time step equal to the mean escape time at each position along the trajectory, on the short 3D fiber model and the short 1D fiber model, produces a ratio of MFPTs 
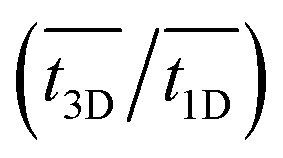
 of unity. Since the MFPT ratio is unity, the finite 3D volume of the cable bacteria conduction channels likely has a small influence on the mobility, leading to an identical range of hopping parameters (seen in Fig. 6) for both 1D and 3D conduction channel cases.”

## 2 Addendum

### 2.1 Summary

The convention used in the original publication for measuring time in the random walks is not suitable for all structures. We used the convention that the time elapsed is *t* = *m*/*k* for *m* steps in a trajectory with hopping rate *k* (in reduced units, where *k* = 1, this amounts to counting steps to measure time). A more correct measure of time is
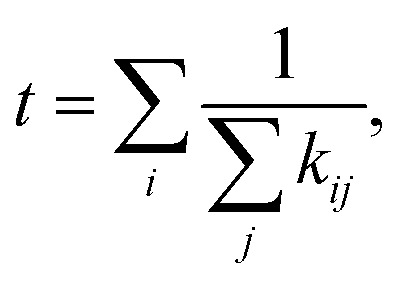
where the sum on *i* runs over particle positions in the trajectory and the sum on *j* runs over the neighbors of site *i* (the mean escape time convention). To describe a system with hopping rates determined by Marcus-like theories, the mean escape time convention is more appropriate because each hopping rate is determined pairwise. Since each hopping rate is independent of the presence or absence of other hops, a particle should reside for a shorter time at a site with more neighbors. Conversely, the step counting method forces a particle to remain at all sites for the same time, regardless of the number of neighbors since Δ*t* is fixed.

The ratios of mean first passage times (MFPTs) for cable bacteria (CB) and a one-dimensional (1D) chain derived from the two time-keeping approaches produce the same result. This occurs because the MFPT in the mean escape time convention (*t̄*_phys_) may be related to the MFPT measured in step counts (*t̄*_steps_) through an effective waiting time *τ*,*t̄*_phys_ = *τt̄*_steps_.The ratio of MFPTs is thus
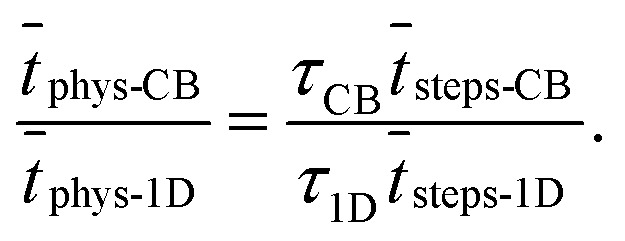
The step-counting and mean-escape time conventions produce the same result when *τ*_CB_/*τ*_1D_ = 1.

For a periodic 1D chain, *τ*_1D_ = 0.5 because each site has two neighbors. In the CB model that was used, each site had two or more neighbors, so *τ*_CB_ < 0.5. However, since most sites in a CB belong to fibers or to the spine (with two neighbors), rather than hubs and branch points with higher coordination, we show here that *τ*_CB_ ≥ 0.49. As
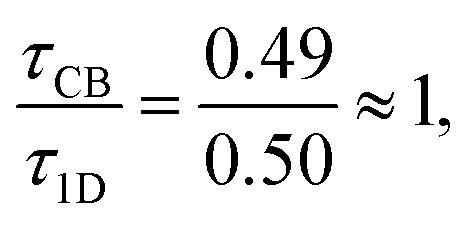
within the ∼3.5% uncertainty of the random-walk simulations, both time conventions produce the same result.

The more general formulation leads to small changes to the interpretation of the simulation data, detailed below. We have also recomputed many of the figures in the original publication using the mean escape time definition, and we find that the two conventions lead to the same conclusions (note that figures are labeled with the same number as in the original submission with an additional “-C” in the correction). The main change to the conclusions in the SI of the paper appears in Section S5, discussed in Section 1.2.2 of this correction. Section S5 reported that the non-1D structure of individual conduction pathways led to a factor of 2.61 increase in MFPT. This finding arose from differences in the effective waiting time and the use of the original step-counting time convention. When the mean escape time convention is used, the MFPT remains unchanged when the possible three-dimensional structure of individual conduction channels is considered, although the 1.5 fold increase in MFPT discussed in the main text, arising from the effect of junctions on multiple conduction channels, remains unchanged.

### 2.2 Primary differences between time-keeping conventions

Although Fig. 5 accurately describes the CB MFPT ratio, we include Fig. 5-C here to validate this result using the mean escape time convention. The figure shows that, when the mean escape time convention is used, the ratio depends on the number of coarse-grained beads used (which is inversely related to the bead diameter). Using 17 coarse-grained beads produces a ratio of 1.46, which lies within the combined error due to window size and the coarse-graining threshold (∼3.5%) of the originally reported value of 1.5. As in the original publication, open circles denote results obtained using a less stringent convergence threshold of *θ* = 0.01.


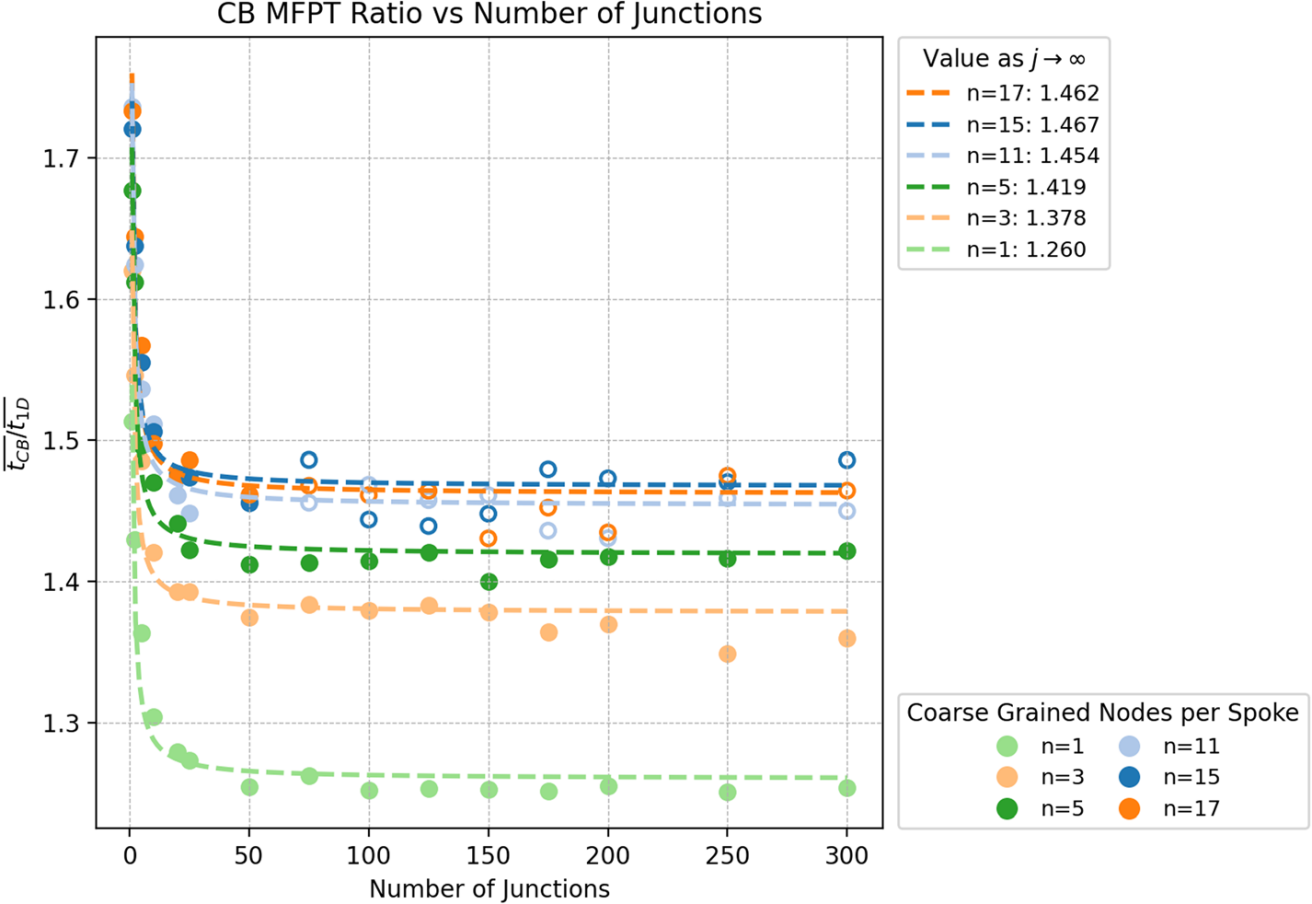
 **Fig. 5-C** The number of coarse-grained sites approaches the number of actual sites (100s–1000s for 1–10 nm cofactor separations), and the ratio of MFPTs approaches 1.5. The ratio converges to within the 3.5% threshold error of the originally reported value of 1.5 at 17 coarse-grained sites.


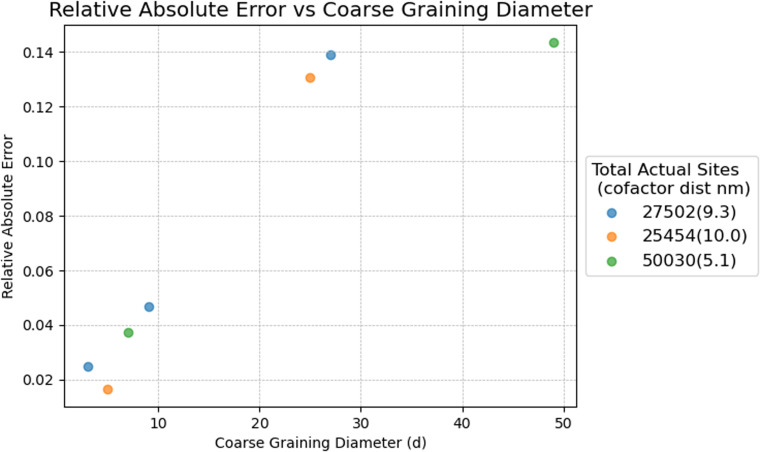
 **Fig. S5-C** In contrast to the originally published step counting time convention (Fig. S5), where the mean escape time convention is used, there is a clear increase in the relative absolute error MFPT in the CB model with increased coarse-graining diameter. Fig. 5-C shows the influence of the number of coarse-graining sites (inversely proportional to coarse-graining diameter) on the ratio of MFPTs.

When the mean escape time convention is used to compute the MFPT in a CB model, the influence of the coarse-graining scale on the MFPT is substantially larger (>10%) than in the original publication (where it was ∼1%). This behavior is expected, since the effective waiting time depends on the distribution of coordination numbers in the CB model. The fraction of sites in a CB model with two neighbors varies with the coarse-graining diameter: decreasing the diameter increases the number of sites with two neighbors within each fiber and spoke, while the number of hubs and branch points (which have more than two neighbors) remains unchanged. One therefore needs to choose the coarse-graining scale to ensure that the number of neighbors assigned to each node in the coarse-grained model appropriately represents the number of neighbors each node has in the full system, thereby minimizing coarse graining error (see Fig. 5-C).

In the SI Section S4A, the ratio of MFPTs, 
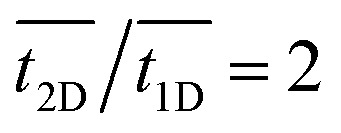
, only holds when the old step counting time convention is used. When the new mean escape time convention is used, this ratio is 
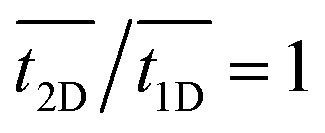
. Using the mean escape time convention results in Fig. S4-C.


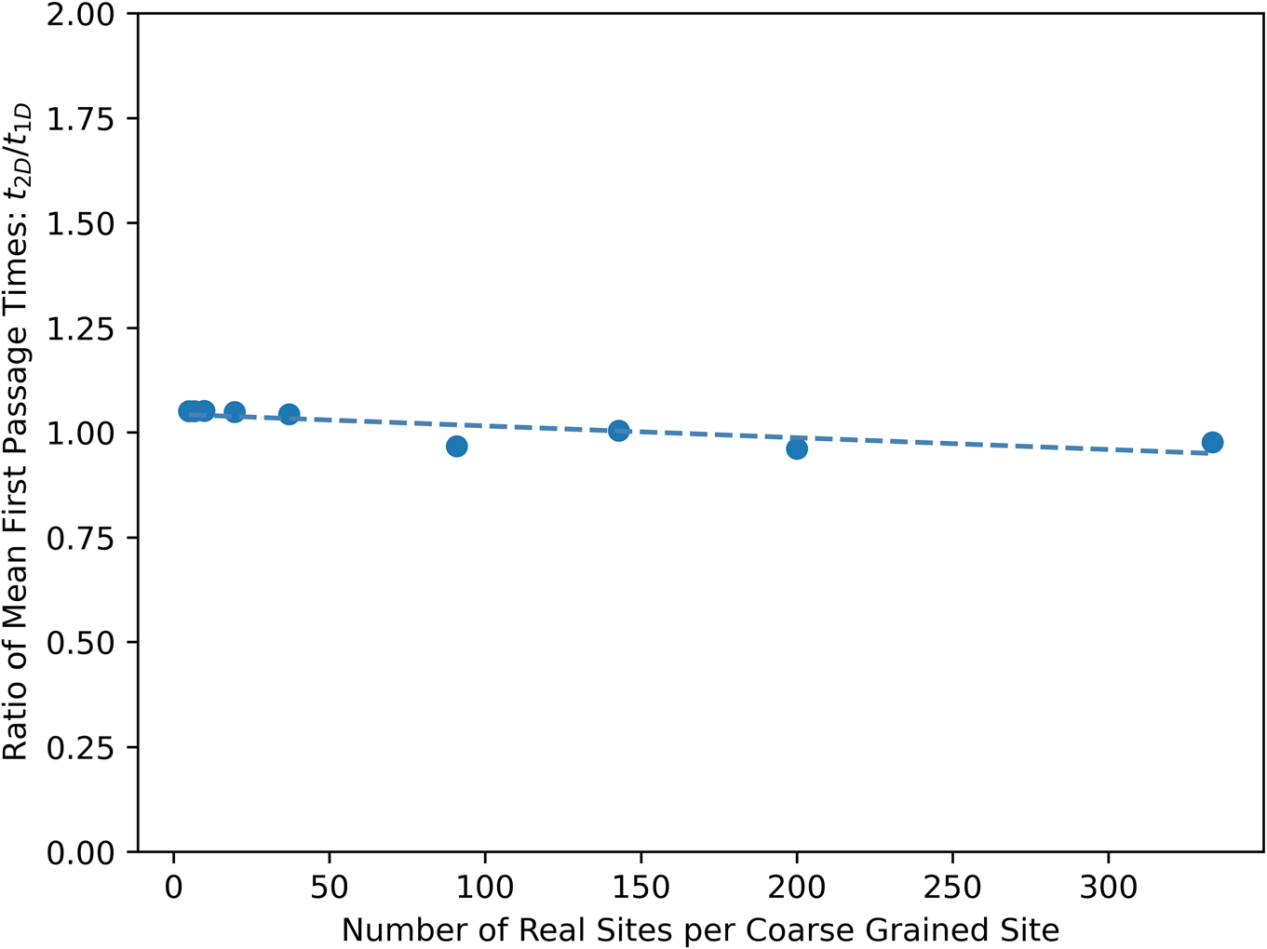
 **Fig. S4-C** The (analytically) expected ratio of the MFPT on a 2D grid to the MFPT on the 1D chain of the same length is equal to one for the mean escape time convention. The influence of coarse-graining on the computed MFPTs is likely much smaller than the influence of sampling noise for uniform lattices (based on MFPTs that are converged to 5% relative standard deviation). For lattices where sites may have different numbers of neighbors, the coarse graining may have a larger effect on the relative absolute error in MFPTs, see Fig. S5-C.

### 2.3 Minor differences between time-keeping conventions

#### 2.3.1 SI Sections S1 and S3A

The discussion in Sections S1 and S3A is based on the step-counting time convention and, therefore, the findings differ from results obtained using the mean escape time convention by a factor of 1/2. This difference does not change the conclusions of these sections.

#### 2.3.2 Mean escape time version of Fig. S2 and S3

The mean escape time versions of Fig. S2 and S3 differ quantitatively from those published in the original SI, but the conclusions remain qualitatively unchanged. Since Fig. S2 and S3 describe small errors arising from the window size and convergence threshold used in the random-walk simulations, the modest quantitative differences are likely due to statistical variation in the random trajectories (produced by different numbers of sampled trajectories that yield the same MFPT).


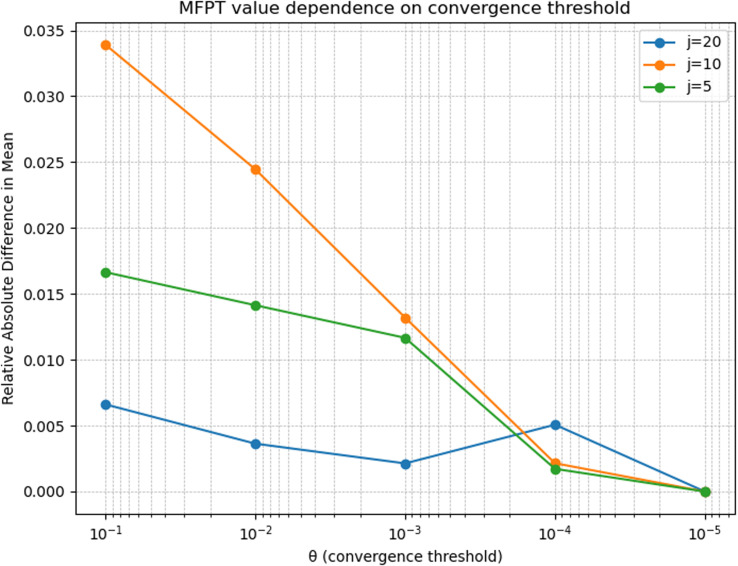
 **Fig. S2-C** Although a clear correspondence between the number of junctions (*j*) and the magnitude of the error is not found, this figure (and the originally published Fig. S2) both indicate that errors due to changes in the convergence threshold (*θ*) are less than ∼3.5%.


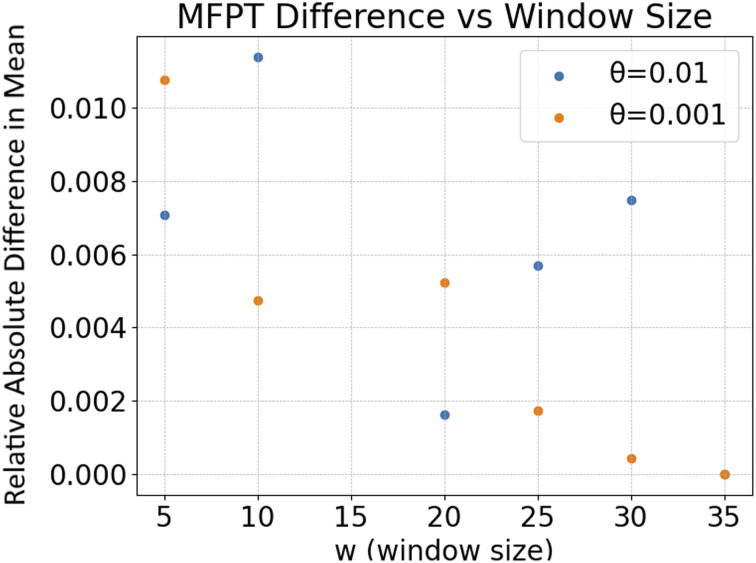
 **Fig. S3-C** Although neither Fig. S3 nor Fig. S3-C indicate a clear correspondence between window size and error, both this figure and the originally published Fig. S3 indicate that errors due to changes in window size are less than 1.3%.

The Royal Society of Chemistry apologises for these errors and any consequent inconvenience to authors and readers.

