# Supplementary material for: Correction: A theoretical framework to understand high electron mobilities in cable bacteria
Source: Chem Sci. 2026 Mar 31;17(16):8305–10. doi: 10.1039/d6sc90073a (PMC13037506; doi:10.1039/d6sc90073a)
Supplement: SC-017-D6SC90073A-s001 [file SC-017-D6SC90073A-s001.pdf]

Supplementary Information (SI) for Chemical Science.
This journal is © The Royal Society of Chemistry 2026

A theoretical framework to understand high electron mobilities in cable bacteria

Andrew J. Smith[a] and David N. Beratan[a,b,c]

[a] *Department of Chemistry, Duke University, Durham, NC 27708, United States.* [b] *Department of Physics, Duke University, Durham, NC 27708, United States.* [c] *Department of Biochemistry, Duke University, Durham, NC 27710, United States*

E-mail: andrew.smith@duke.edu, david.beratan@duke.edu

S1 The relationship between mean first passage time ratios and mobilities for cable bacteria

This section motivates eqn (3) of the main text, linking ratios of mean first passage times (MFPTs) to mobilities. In d-dimensional isotropic systems, the diffusion coefficient is proportional to the mean-squared displacement. The diffusion constant can be written as the sum of mean-squared displacements in each direction:[1]

$$\langle r_d^2 \rangle = \sum_{i=1}^{d} \langle {x_i}^2 \rangle = 2dDt \tag{S1.1}$$

Here, $\langle r_d^2 \rangle$ is the mean-squared displacement in d-dimensions, $\langle {x_i}^2 \rangle$ is the mean-squared displacement in dimension i, D is the diffusion coefficient, and t is the time. t is taken to the infinite limit, and the diffusion coefficient is defined as being proportional to the mean-squared displacement over the elapsed time t:[1,2]

$$D = \frac{1}{2dt} \langle r^2 \rangle \tag{S1.1.1}$$

For an *anisotropic* medium, we expand the sum in eqn (S1.1) as a sum of distinct directional diffusion coefficients:[1]

$$\langle r_d^2 \rangle = \sum_i^d \langle {x_i}^2 \rangle = \sum_i^d 2D_i t \tag{S1.1.2}$$

If $D_i = D$ for all i, the sum in eqn (S1.1) is recovered. eqn (S1.1.2) follows from the one-dimensional diffusion relation for direction i:[2,3]

$$D_i = \frac{1}{2t} \langle {x_i}^2 \rangle \tag{S1.1.3}$$

We focus on the electronic mobility in direction i between the source and drain electrodes in our study. The Einstein-Smoluchowski relation describes the mobility as being proportional to the diffusion constant divided by temperature:[4]

$$\mu_i = \frac{qD_i}{k_B T} \tag{S1.2}$$

where μ_i is the electronic mobility in direction i, q is the charge of the moving particle, k_B is Boltzmann's constant, and T is the temperature. The ratio of mobility in a 1D chain to the mobility in the CB model with junctions (taking the mobility in the direction of current, i.e., parallel to the fibers) is:

$$\frac{{\mu_i}^{1D}}{{\mu_i}^{CB}} = \frac{q{D_i}^{1D} k_B T}{k_B T q {D_i}^{CB}} = \frac{{D_i}^{1D}}{{D_i}^{CB}} \tag{S1.2.1}$$

In the main text, for ease of computation, we compute the mean first passage time (MFPT) given by:[2,3]

$$\overline{t_i} = \frac{1}{2D_i} {x_i}^2 \tag{S1.3}$$

for a single dimension.

The ratio of MFPTs for CB and 1D chains is:

$$\frac{\overline{{t_i}^{CB}}}{\overline{{t_i}^{1D}}} = \frac{2{D_i}^{1D} {x_i}^2}{2{D_i}^{CB} {x_i}^2} = \frac{{D_i}^{1D}}{{D_i}^{CB}} \tag{S1.3.1}$$

Since the right-hand side of eqn (S1.2.1) equals the right-hand side of eqn (S1.3.1) , we have (from the main text):

$$\frac{\overline{t_i^{CB}}}{\overline{t_i^{1D}}} = \frac{\mu_i^{1D}}{\mu_i^{CB}} \tag{S1.4}$$

This result indicate that the ratio of MFPTs for two systems is equal to the reciprocal of the ratio of the systems' mobilities.

S2 Statistical analysis of cable bacteria conductivity data

This section describes the challenge of modeling electrical conductivities and mobilities in CB, given the wide range of reported structures and conductivities.

S2A Conductivity spread and focus on mobility

The scarce CB mobility data for the *Ca. Electrothrix Communis RB* species of CB are narrowly distributed around a mean value of 0.19 cm^2/Vs (there are 4 reported mobilities ranging from 0.09 to 0.27 cm^2/Vs).[5] While there are significantly more measurements of CB conductivities (as many as 270 measurements across all species and all measurement methods - see Table S1), the difference between minimum and maximum conductivity values spans 4 (grouping i) to 11 (grouping a) orders of magnitude, depending on the data considered (grouping d spreads over 7 orders of magnitude - see table S2) . This spread of the data presents a theoretical challenge, as assessing a model as being compatible or incompatible with this conductivity data depends on which points are modeled. For example, one might expect that the minimum (2.46×10^{-5} S/cm in group d) and maximum (546 S/cm in group d) are produced by different mechanisms. Rather than consider the wide range of mechanisms that could arise from the broad spread conductivity data, we instead analyze the electronic mobilities. A number of explanations for the wide variance in the CB conductivity data are plausible: variation in CB diameters,[6,7] growth conditions,[8–11] oxygen exposure,[8–11], or damage to samples during fiber sheath preparation.[8,9,11,12] While these issues may influence the

group letter	group description	sample size	mean	median	standard deviation
a	All data points (excluding the averages of already included replicas)	270	8.35	1.10	39
b	All data points representing replicas by their average	252	8.82	0.95	40.3
c	All data points, excluding total conductivity from protonic conductivity experiments (and excluding the averages of already included replicas)	249	9.05	1.60	40.5
d*	All data points, excluding total conductivity from protonic conductivity experiments, representing replicas by their average	231	9.62	1.41	42
i	4 probe Rattekaai measurements (excluding the averages of already included replicas)	23	11.61	1.75	19
j	4 probe Rattekaai measurements, representing replicas by their average	15	17.21	10.00	21.7
k	Rattekaai 2 probe measurements (excluding the averages of already included replicas)	160	11.55	2.01	49.7
l	Rattekaai 2 probe measurements, representing replicas by their average	150	12.18	1.68	51.3
t	all Electrothrix communis RB	17	0.13	0.10	0.154

Table S1: Summary Statistics for Select Literature Conductivities. All values are in S/cm (except sample size, which is a unitless number). The table shows summary statistics for selected groupings of literature data. Additional data summary statistics and grouping methods appear below. The jump in group letters makes it clear that this is not an exhaustive list of all the groupings we considered, but is rather an illustrative subsample. *Statistics for group d are referenced in the main text.

group letter	relative standard deviation	min	max	(Q1-Min)/IQR	(Max-Q3)/IQR
a	4.67	1.23e-09	564	0.0235	126
b	4.57	1.23e-09	564	0.0183	106
c	4.47	2.46e-05	564	0.0371	106
d*	4.36	2.46e-05	564	0.0233	89.5
i	1.64	0.31	74	0.0469	4.03
j	1.26	0.63	74	0.0473	2.17
k	4.31	0.0034	564	0.035	75.2
l	4.21	0.0034	564	0.0297	66.1
t	1.16	0.000282	0.553	0.199	2.71

Table S2: Selected Distribution Descriptors. *Columns "min" and "max" are in S/cm. All other columns are unitless.* A description of what the group letters correspond to is found in table S1. Note that the group letters being non-sequential indicates that this is only an illustrative subsample of all the data groupings we considered (the full set can be found in the accompanying spreadsheet "Supplement_fullLitData.csv"). The relative standard deviation is calculated as the standard deviation divided by the mean. The last two columns indicate how far the extrema of the distribution are from the nearest quartile to show that a few points may have a dramatic effect on the mean. *Statistics for group d are referenced in the main text.

conductivities, it is unclear whether these factors can account for the 7 orders or more of variation in the observed conductivities.

S2B Method of analyzing conductivity statistics

In the main text, (and below, in the supporting information), we present summary statistics and distribution descriptors for the CB conductivities (see tables S1 and S2, and the accompanying spreadsheet "Supplement_fullLitData.csv"). We included all published peer-reviewed conductivity data available at the time of submission (see references[5,7,8,10,12–14]). These summary statistics depend on which literature data are included in the analysis. As shown in table S1, the reported conductivities vary with the experimental methods (2 probe vs 4 probe), the source of the CB, and the species of CB. The large variance in conductivities between species and sample collection sites may reasonably lead one to conclude that only samples from a single source (e.g., Rattekaai) should be included in the data analysis. However, some data[7] suggest that CB have broadly similar metallic compositions and that conductivities associated with different sample locations may not differ

in a statistically significant way. As such, one could reasonably suggest that the charge-transfer mechanism is conserved across CB, and that all data from all sampling locations and species should be included in the statistical analysis. As both of these interpretations are reasonable - but contradictory - we believe choosing one strategy over the other would add subjectivity to the analysis. To avoid this, we compute summary statistics for over 20 different ways to represent the literature data (see "Supplement_fullLitData.csv"). Choices about data inclusion influence the interpretation of the statistics. We also considered how best to represent repeated measurements on the same CB specimen. If all CB specimens have the same conductivity mechanism, every individual measurement should be considered equally in the statical analysis. If the mechanisms differ between CB specimens, then one could average repeated measurements on the same physical specimen. This averaging would avoid over representing a single specimen that may have atypical characteristics. For example, the median conductivity value of the 4 probe Rattekaai measurements is 1.75 S/cm if all repeated measurements are included in the statistical analysis. However, the median 4 probe Rattekaai conductivity value is 10 S/cm if repeated measurements on the same physical specimen are averaged and then the single averaged value is used in the subsequent statistical analysis. In order to avoid subjectivity in the treatment of repeated measurements, we report statistics computed by both methods.

S2C Utility of CB median conductivity data

Although we focus on electronic mobility, when conductivity is cited, we refer to the conductivity of a typical CB specimen, namely the median conductivity of the CB, regardless of species or source. We use the median conductivity rather than the mean conductivity to better represent a typical CB, as some CB are much more or less conductive than others. When discussing group d, it is important to note that this group excludes proton conduction experiments of Ref.[13]. Although the proton conductivity experiments do measure a total conductivity (which includes both electron and proton contributions)[13 13]. However, these experiments use significantly different techniques to measure conductivities compared to experiments that focus on the electronic conductivity. As such,

the conductivities of the two kinds of experiments may not be comparable. In group d, we average repeated measurements on the same CB specimen before the final statistical analysis. However, comparing the data of group d to that of group c (which includes each individual measurement, rather than their average value) shows that - for this specific data set - either way of representing the repeated measurements produces similar results.

In most discussions of the conductivity (in the SI and main text), we focus on median values. The median values rather than the mean values better represents a typical CB, because the vast majority of CB data groupings have points far above the 3rd quartile. The IQR (interquartile range) value is a common statistical metric to identify outlying data.[15–18] Data beyond 1.5·IQR above the 3rd quartile are frequently considered to be outliers.[18] As seen in table S2, most strategies to group the data produce maximum values well above 1.5·IQR above the third quartile, with many (including grouping d) having points more than 50·IQR above the third quartile. This finding suggests that a few extreme points may dramatically increase the mean conductivity value. This increase in the mean conductivity, relative to the median conductivity, is not a reflection of the conductivity of a typical specimen. Rather the mean is strongly influenced by high conductivities of a few points. Regardless of which data are included in the calculation, the median remains largely unchanged - with values around $\sim 10^0$ S/cm. A notable exception occurs for group j, where the median is 10 S/cm. As group j includes 4 probe measurements, these values may indicate that the Rattekaai CB be different (more conductive) than the freshwater CB and CB from other locations. However, reaching the conclusion that the Rattekaai CB may have a different charge transport mechanism depends upon how repeated measurements on the same sample are treated (groups i vs j). For these reasons, we focus on group d data.

The largest CB conductivity data points (> 500 S/cm) would qualify as being outliers. Nonetheless, the largest conductivity data are not necessarily non-physical or erroneous. Rather, the outlying conductivity measurements are atypical. While the difference in conductivity between typical and outlying measurements may arise from unknown sources of error, these differences may also arise from a different conductivity mechanism in the outliers. Many factors influence the conductivity

(see section S2A), but our understanding of their influence remains qualitative. As such, rather than focusing on a maximum hypothetical conductivity of CB, we choose to focus on the conductivity values of typical CB.

S3 Convergence of computed MFPTs for CB

We now motivate the choice of convergence criteria used for the random walk simulations of MFPTs in CB. We also discuss the influence of convergence hyperparameter choices on the computed MFPTs for CB.

The converged MFPT is the sum of the probability-weighted first passage times over infinitely many trajectories (the MFPT is approximate for a finite number of trajectories):

$$\overline{t_{mfp}} = \langle t_i \rangle_{n=\infty} = \sum_{i=0}^{\infty} p_i t_i \tag{S3.1}$$

t_i is the ith first passage time, and p_i is the probability of a particle taking path i . We compute the first passage times for 100 independent random walks between chain ends. The mean of these first passage times (the sample MFPT - denoted $\langle t_i \rangle_{n=100}$) approximates the converged MFPT. To better approximate the converged MFPT, we use the central limit theorem. This theorem prescribes that when one averages over many ($m \to \infty$) sample means (samples of size n with mean $\langle x \rangle_n$), the difference between the mean value of the sample means ($\langle \langle x \rangle_n \rangle_m$ and the true population mean μ_{pop}) approaches zero:

$$\lim_{m \to \infty} (\langle \langle x \rangle_n \rangle_m - \mu_{pop}) \to 0 \tag{S3.1.1}$$

For approximate MFPTs, we denote the mean of the sample means over m samples of size n each as $\langle \langle x \rangle_n \rangle_m$. We can recover the converged MFPT ($\langle t_i \rangle_{n=100}$) in the limit of $m \to \infty$:

$$\epsilon = \langle \langle x \rangle_{n=100} \rangle_m - \langle t_i \rangle_{n=100} \tag{S3.1.2}$$

$$\lim_{m \to \infty} (\epsilon) \to 0 \tag{S3.1.3}$$

eqn (S3.1.2) defines the difference between the MFPT (approximated by averaging over m samples of size $n = 100$) and the converged MFPT. Since we cannot reach the infinite m regime numerically, we must assess when the convergence of the mean of sampled MFPTs is reached, where the value of ϵ approaches zero. Since ϵ becomes arbitrarily close to zero as the number of samples m approaches infinity, we expect ϵ's standard deviation to shrink (since the converged MFPT is a constant, the standard deviation of ϵ is determined entirely by the mean of MFPTs). We determine the convergence of ϵ to be reached when the relative standard deviation of the mean of sample MFPTs (σ_{sMFPT}) is less than the threshold value $\theta = 0.001$. As we focus on the value of σ_{sMFPT} in the converged limit, we compute σ_{sMFPT} over the last $w = 20$ samples - w defines our window size. Computing the actual value of the mean of MFPTs requires averaging over all samples - even those outside of the window. Next, we explore the influence of choosing different values for θ and w, and we demonstrate that the influence of these values on the mean of sampled MFPTs is much smaller than the CB dimensionality effects on the approximated converged MFPT (which determines mobilities) that we discussed in the main text. We refer to the relative absolute difference (RAD) below, namely the absolute difference in the outcome of a MFPT calculation performed with a lower level of certainty, $MFPT_L$ (a lower level of certainty due to the smaller θ or w values) and a calculation performed with a higher level of certainty, $MFPT_H$, divided by the higher certainty result:

$$RAD = \frac{|MFPT_L - MFPT_H|}{MFPT_H} \tag{S3.2.0}$$

S3A Validation of MFPT calculations on a 1D chain

The MFPTs computed using the 1D chain model agree within 1% of the expected analytical result (Fig. S1). Errors in the MFPT are not systematic over or underestimates, suggesting that they originate from the finite convergence threshold and not from systematic errors. For the comparison

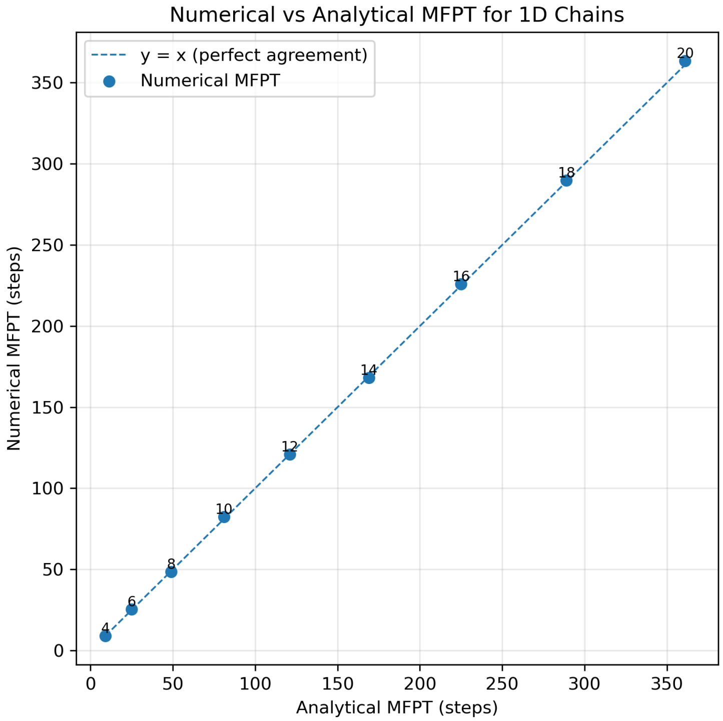

Figure S1: Comparison of the MFPT calculated for a random walk with window size 25 and convergence threshold 0.0001 for various test lengths. All of the points agree with the analytical result, with less than 1% error.

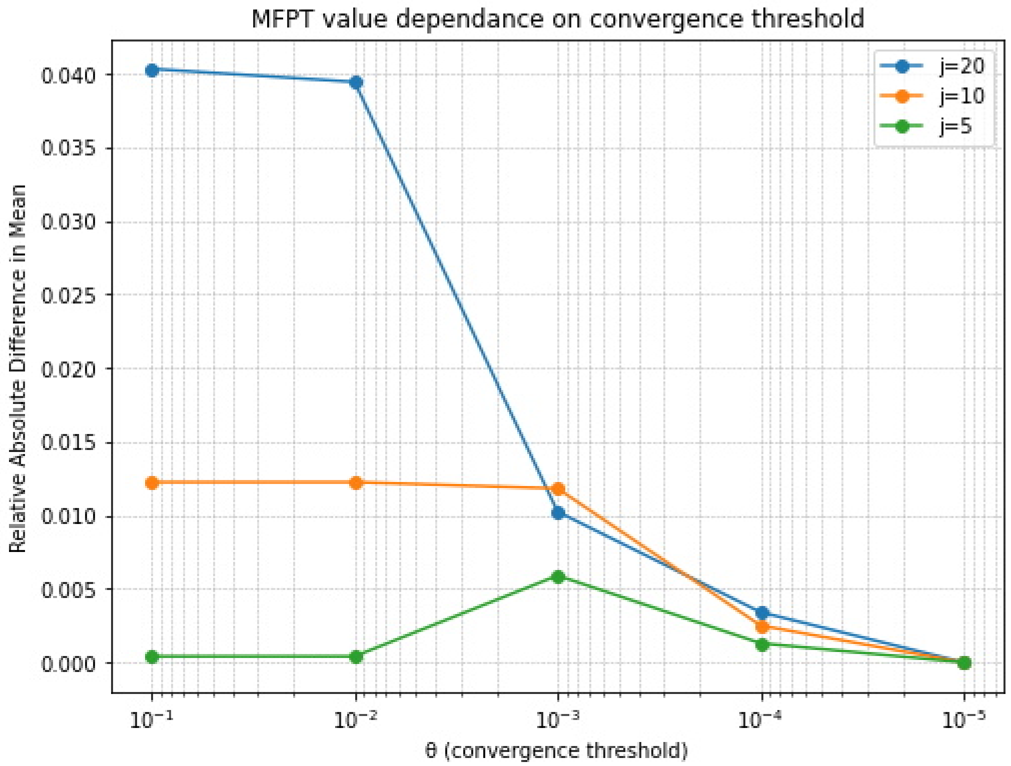

Figure S2: The RAD (relative to the point with the lowest convergence threshold) as a function of the convergence threshold (θ). The data indicate that the error relative to a two orders of magnitude lower value of θ is less than 1.5% for θ=0.001 - which was used in the main text. Here, j in the key represents the number of junctions.

of CB MFPTs to 1D chain MFPTs described in the main text, we used analytically computed 1D MFPTs. The small threshold value for the 1D chain MFPT convergence is computationally tractable for these test chains, but we also examined the influence of different convergence thresholds for the MFPTs for larger systems in the SI section S3B. These 1D chain calculations were performed using a step time counting method, which produces MFPTs that differ by a factor of one-half from the expected mean escape time MFPTs. The points in Fig. S1 show the 1D chain length $L = x + 1$, where x is the total displacement.

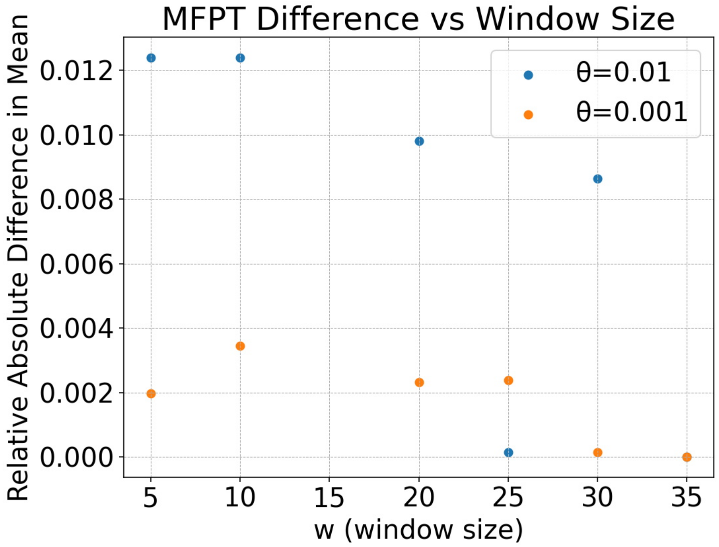

Figure S3: The RAD (relative to the point with the largest sampling window) is shown as a function of the window size w. The errors are small for all values of w (compared to the errors shown in Fig. S2 for the same value of θ). The large variation of the RAD in MFPTs, and the small magnitudes of the RAD in MFPTs, suggest that these differences may arise from stochastic effects on the value of the MFPT, rather than being caused by the value of the window size.

S3B Dependence of the mean of sampled MFPTs on convergence thresholds

The convergence threshold value θ=0.001 (used in calculations of the main text) generally produces small errors in the mean of sampled MFPTs compared to more strictly converged calculations - with errors less than 1.5%(Fig. S2). Although there are large differences in the errors, depending on system size (number of junctions), $\theta \geq 0.01$ produces errors that are similar for all system sizes when $\theta \leq 0.001$, and the errors no longer correlate with the system size for $\theta \leq 0.001$.

S3C Dependence of the mean of sampled MFPTs on window size

The RAD in the MFPT is small compared to the difference (see Fig. S3 and Fig. S2). The differences found for θ=0.001 in Fig. S3 are smaller and have greater variation in the RAD compared to the RAD due to different values of θ seen in the Fig. S2. As such, the RADs in Fig. S3 do not depend

on w, but rather originate in stochastic differences in the MFPTs based on the value of θ. When $w > 10$, the RAD is $< 1\%$, which is much smaller than the error due to any other effect.

S3D Convergence parameters used in the MFPT analysis

All calculations of MFPTs in the main text used $\theta = 0.001$ and $w = 20$, with 60 fibers and a coarse-graining diameter of 501 physical redox sites, unless otherwise noted. Discussion of the coarse-graining diameter can be related to a coarse-graining radius r using $diam = 2r + 1$. Fig. 4 of the main text uses closed circles to indicate the convergence criteria noted above. In that figure, the open circles indicate more relaxed convergence criteria, chosen for computational expediency. This more relaxed convergence criterion is $\theta = 0.01$ with a coarse-graining diameter of 1,001 physical redox sites.

S4 MFPT sensitivity to coarse graining diameter

This section explores the sensitivity of the coarse-grained analysis to the coarse-graining radius. Section S4A explores the effects in a 1D periodic chain and a 2D square lattice, where MFPT results are known analytically. Section S4B shows that the coarse-graining method is exact for 1D periodic chains. Section S4C shows that coarse-graining provides an excellent approximation for the branched CB system.

S4A 1D vs 2D Systems with well-known MFPTs

MFPTs for cubic lattices with identical nearest neighbor hopping rates scale as $t_{mfpt} \propto \frac{1}{2d}$ (where d is the system dimensionality).[19] The MFPTs that we refer to here arise for transport in a single direction (i.e., the current direction). To test our numerical methods, we compare MFPT results for 1D and 2D lattices. The ratio of the MFPTs for 1D and 2D lattices is equal to 2, when using $t = m/k$ for a number of total steps m and a uniform hopping rate k. Note that if the mean escape time at each point is used instead, the expected ratio is unity and the dimensionality scaling of

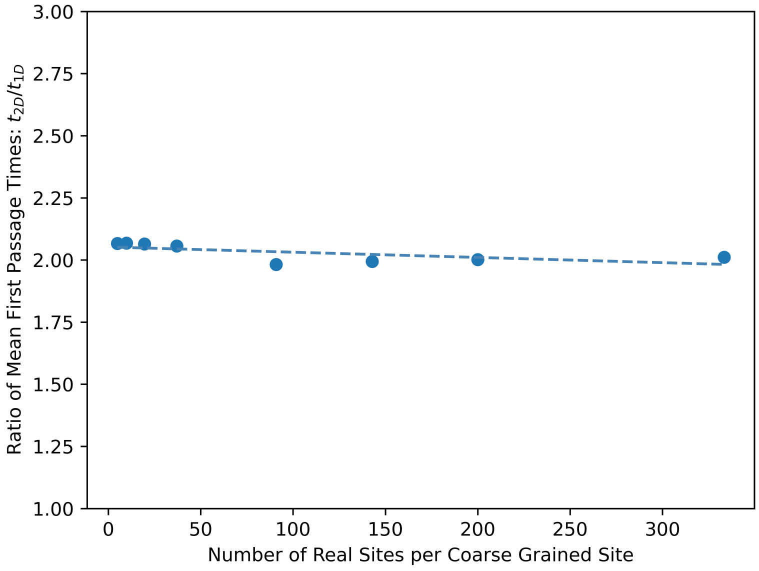

Figure S4: The ratio of MFPTs for a 2D lattice and a 1D chain is influenced by coarse-graining. The analytically expected ratio of the MFPT on a 2D grid to the MFPT on the same length 1D chain is two. The influence of coarse-graining on the computed MFPTs is likely much smaller than the influence of sampling noise (based on MFPTs that are converged to 5% relative standard deviation).

diffusion in a fixed direction vanishes. The results in Fig. S4 are converged to 5% relative standard deviation. Fig. S4 shows that the ratio of MFPTs in 2D to 1D lattices is independent of the coarse-graining diameter, because the ratio of MFPTs fluctuates tightly around the value two (with all ratios within 5% of two) for all choices of the coarse-graining diameter. In both 2D and 1D lattices, the MFPT was computed for a particle to traverse a distance of 11 lattice sites along the x-axis.

S4B Validity of the coarse graining approach for computing MFPTs in 1D networks

The MFPT simulations count the particle's position and the number of hops (which is proportional to the time elapsed). We use units of $time = number\ of\ hops \times\ rate^{-1}$ and $distance = x_0 - x_f$ (where x_0 and x_f are the initial and final particle positions, respectively) along a given transit direction. In these units, the diffusion coefficient is 1/2 in units of hopping time^{-1} hopping distance^{-2}. The resulting MFPT expression for a 1D chain is:

$$\overline{t_{mfp}}^{a} = (x^a)^2 \tag{S4B.1}$$

x is the particle displacement and the superscript a indicates a displacement over physical sites. If a system can be successfully coarse-grained using the procedure in the main text (Section 2.3, then we need to be able to decompose the physicaldistance x^a into a number of coarse-grained sites (A), where each coarse-grained site has the diameter B. For coarse-graining to be physically meaningful, we require that there is an integer number of coarse-grained sites (A), that each coarse-grained site contains an integer number of physical sites (B), and that the total number of physical sites represented by course-gained sites is the same as the number of physical sites in the full system ($x^a = AB$). With these conditions, we can write eqn (S4B.1) as :

$$\overline{t_{mfp}}^{a} = A^2 B^2 \tag{S4B.2}$$

We compute the MFPT for the coarse-grained model of the 1D system using eqn (S4B.1) :

$$\overline{t_{mfp}}^{CG} = A^2 \tag{S4B.3}$$

In eqn (S4B.3) , the CG superscript refers to a MFPT for a coarse-grained distance. Multiplying this expected number of coarse-grained hops by the expected number of physicalhops per coarse-grained hop, we obtain the expected number of physicalhops ($\overline{t_{mfp}}^{a}$). The expected number of physicalhops associated with coarse-grained hops between sites with diameter (B) is:

$$\overline{t_{mfp}}^{a/CG} = B^2 \tag{S4B.4}$$

The superscript a/CG indicates that this is the number of physicalhops for one coarse-grained unit. Multiplying eqn (S4B.3) and eqn (S4B.4) , we recover eqn (S4B.2) :

$$\overline{t_{mfp}}^{CG} \cdot \overline{t_{mfp}}^{a/CG} = A^2B^2 = \overline{t_{mfp}}^{a} \tag{S4B.5}$$

As such, the MFPT computed using coarse-graining is exact in 1D.

S4C Influence of coarse graining the structure of the CB transport networks on MFPT

We now describe CB models with 20 junctions and 8 fibers for both the coarse-grained and full systems MFPT analysis. These models describe structures smaller than those of biological CB and allow us to assess the validity of the coarse-graining approximations. These smaller model systems contain 27,502 physical sites (54 physical sites on each spoke with an inter-site distance of 9.3nm), a model with 25,454 physical sites (50 physical sites per spoke and inter-site distances of 10 nm), and a model with 50,030 physical sites (98 physical sites per spoke and inter-site distances of 5.1 nm). In all of these models, the fundamental CB topology (consisting of fibers, junctions, and spokes - as discussed in main text Section 2.1) remained unchanged. The lengths of the fibers, junctions, and spokes also remained the same as described in the main text Section 2.1. For these validating

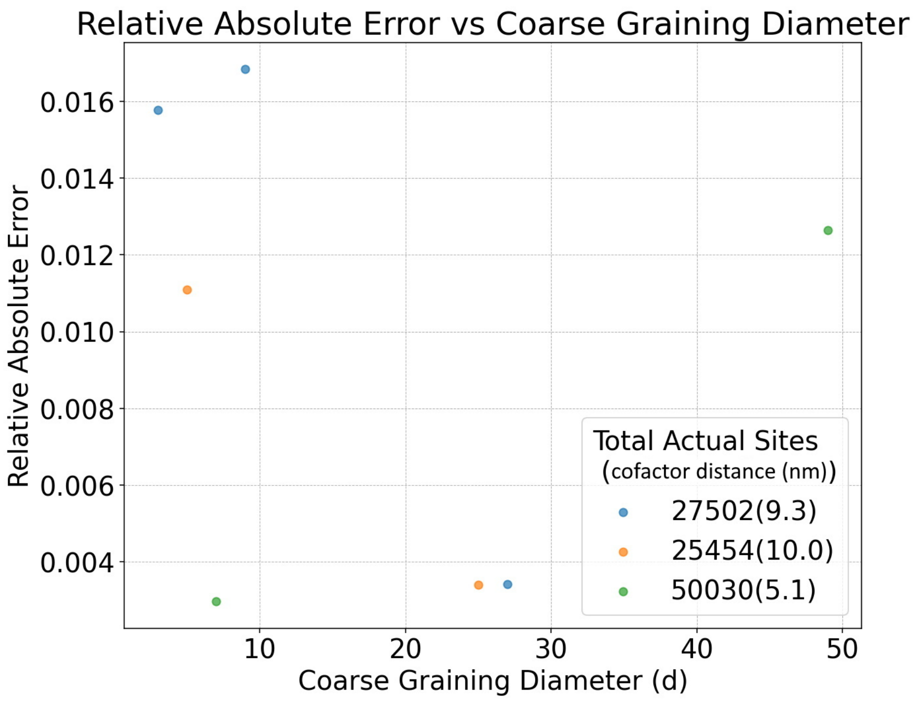

Figure S5: The relative absolute error in the computed MFPT for CB models as a function of the coarse-graining diameter. The plot indicates that there is no clear correspondence between the error and either the coarse-graining diameter or the system size. The relative absolute error in MFPTs for the coarse-grained system compared to the full system is always less than 2%.

calculations, we changed the distance between hopping sites (which also changes the total number of sites in a fixed physical distance). Decreasing the number of sites provides computational ease, and the total number of sites must be divisible into an integer number of coarse-grained sites to apply the coarse-graining methodology. Furthermore, the total number of sites must be divisible into different numbers of coarse-grained sites (with each division corresponding to a unique coarse-graining diameter) to be able to study the effect of changing the coarse-graining diameter on the MFPT. The large inter-cofactor distances used for these validation calculations have no direct link to experiments - they provide a model structure to investigate the influence of different coarse-graining diameters on the calculated MFPT. The validation calculations demonstrate that the relative absolute error was consistently less than 2%. Although these calculations were simplified from the perspective of the structure of biological CB, the absence of correlation between the number of physical sites or the coarse-graining diameter suggests that errors are likely to be similarly small for much larger calculations.

S5 Assessment of how the conducting fiber model influences the computed MFPTs

This section explores the ability to describe transport through individual conducting fibers using 1D hopping chain models. CB fibers are much larger (>20 nm in diameter[6]) than the conduction channels of other biological conductors, like bacterial nanowires (<4 nm in diameter[20]). While bacterial nanowire conduction is usually attributed to single 1D chain of heme cofactors, the CB size allows for more complex transport networks. Prior studies[11] modeled the structure of fibers as a series of tightly packed 1D chains of redox active units. However, there is limited structural information on the fibers. Imagining[7] supports the presence of multiple 1D chains within a fiber; the resolution of this data is not sufficient to determine the placement or orientation of redox active units. If the redox sites are confined to the interior of each chain that make up each fiber, the chains can likely be treated as non-interacting conducting chains (as shown in Fig. S6B). If fibers

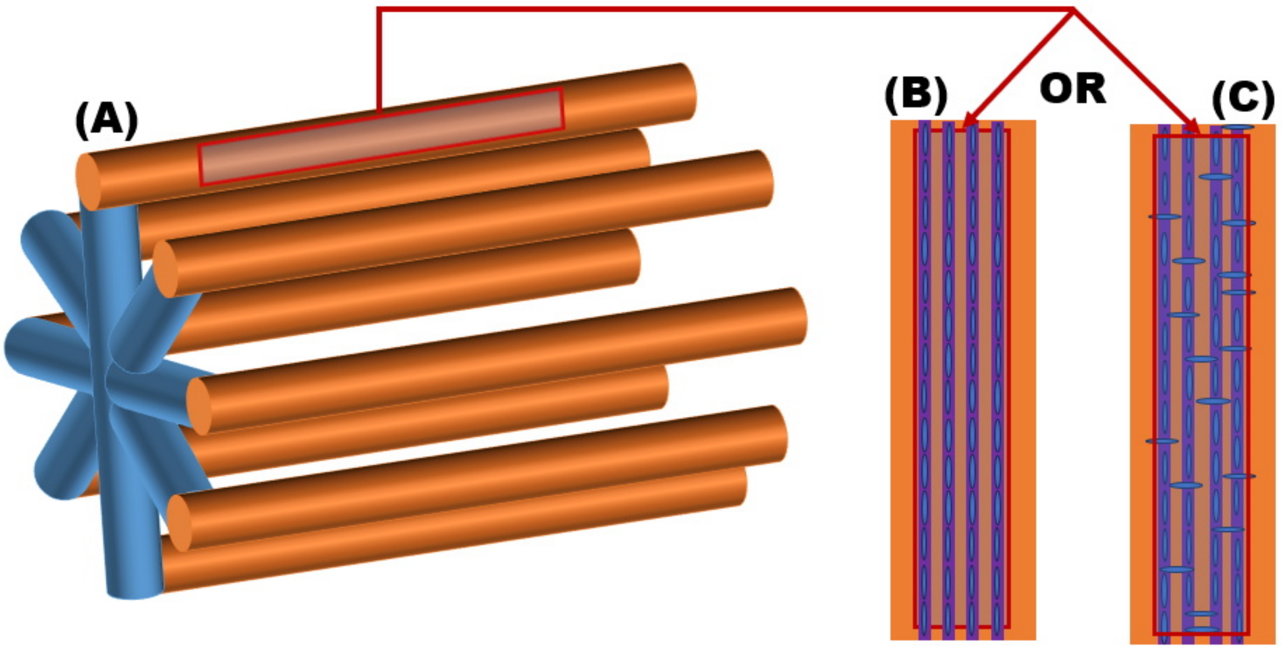

Figure S6: The fibers in CB could be built of either non-interacting 1D chains (B) or interacting chains (forming a 3D lattice) (C) - which may be represented by a higher-dimensional lattice. Panel (A) shows a schematic CB as described in main text Fig. 1. The red box shows where a cross-section of the fiber is taken. Panels (B) and (C) show the zoomed-in cross section and the 1D chains (purple), which experiment indicate[7] constitute the CB fibers. Panel (B) shows that the 1D fibers are made up of tightly aligned and confined redox sites (blue ovals), constitute non-interacting 1D chains. Panel (C) shows that if the redox sites are not tightly confined, or lie on the edge of the 1D chain, this can create interactions between chains that are not captured in a simple 1D model.

are comprised of non-interacting transport chains, the MFPT analysis in the main text accurately describes the electronic mobilities. Dimensionality is known to influence mobility, so if the fibers consist of interacting chains (Fig. S6C), the mobility of non-interacting chains would be an over estimate (see main text Section 2.2). Here, we examine the extreme case of representing fibers as consisting of a 3D cubic lattice (to represent a highly interconnected network of hopping chains). We find that our model 1D chain analysis qualitatively reproduces the results for 3D lattices.

To explore the nature of the 3D cubic lattice model, we compare the ratio of the mean first passage times for 1D lattices compared to finite width and height 3D cubic lattices. We selected a cutoff length of 2,000 sites and a width/height of 5 sites. These dimensions were chosen to model fibers that are much longer than they are wide, corresponding to CB.[9] Assuming that the conductive fiber is confined to a $\sim 20\ nm$ diameter cylinder (motivated by structural data)[6], a small CB (only $8\ \mu m$ long) would be 400 times longer than it is wide (which is why $2,000/5 = 400$ was chosen). Such a small CB structure has a lower length-to-width/height ratio than a typical experimental specimen on the $100s\mu m$ to cm scale. The MFPT and electrical mobility of a model short 3D CB fiber will have a higher MFPT and lower mobility than for an experimentally sized longer 3D CB fiber. Performing an unbiased random walk with a time step equal to the mean escape time at each position along the trajectory, on the short 3D fiber model and the short 1D fiber model, produces a ratio of MFPTs ($\overline{t_{3D}}/\overline{t_{1D}}$) of unity. Since the MFPT ratio is unity, the finite 3D volume of the cable bacteria conduction channels likely has a small influence on the mobility, leading to an identical range of hopping parameters (seen in Figure 6) for both 1D and 3D conduction channel cases.

S6 Additional Marcus parameter discussion

S6.1 Grid Search details

As described in the main text, we probed the range of Marcus parameters: $0.5 \leq r \leq 2.5\ nm$, $1 \leq H_{DA} \leq 30\ meV$, $0.16 \leq \lambda \leq 0.36\ eV$, and $\Delta G^{(0)} = 0\ eV$. To explore this range of parameters,

we examined 1,160,000 points corresponding to step sizes of $\Delta\lambda = 1meV, \Delta H_{DA} = 1meV, \Delta r = 0.1$Å$= 0.01nm$ (resulting in 1.16 million grid points in parameter space). These step sizes were chosen to be similar to the degree of precision at which these parameters are often reported.[21,22]. To ensure that our result is robust with respect to the number of grid points chosen, we also calculated the proportion of points that agree with the experimental average mobility within one experimental standard deviation and experimental precision using an order of magnitude smaller (1200 points) and an order of magnitude larger (1.16 billion points) step sizes for all parameters. When we decreased the step size (1.16 billion points) 22.59% ($\sim$ 262 million points) were within one standard deviation of the experimental mobility and 2.16% ($\sim$ 25.1 million points) were within the experimental precision of the average experimental mobility. This is similar to the 22.03% ($\sim$255 thousand points) within one standard deviation and 2.11%(24.535 thousand points) within experimental precision observed in the original 1.16 million grid points. When we increased the step size by an order of magnitude (1.2 thousand points), the proportions became 13.5% (162 points) within one standard deviation and 1.5% (18 points) within experimental precision. This suggests that our original 1.16 million point grid was sufficiently fine as the result did not substantially change upon increasing the number of points, but that a coarser grid would have missed significant portions of the parameter space.

S6.2 Discussion of parameters that are inconsistent with experimental mobilities

We discuss analysis of transport for over one million sets of Marcus parameters that were chosen in a range typical for biological structures (in the main text and SI section S6.2). This analysis finds a sizable region of parameter space ($\sim$ 25%) of points that are within 1 experimental standard deviation of the experimental average mobility. This finding suggests that CB mobility can be explained using a biologically typical set of Marcus parameters. We do not expect all of the tested Marcus parameters to produce mobilities close to the experimental values. The full range of mobilities produced is shown in Fig. S7A. Many of the experimentally inconsistent computed mobilities are larger than the experimentally measured mobilities. The parameter distributions that did or did

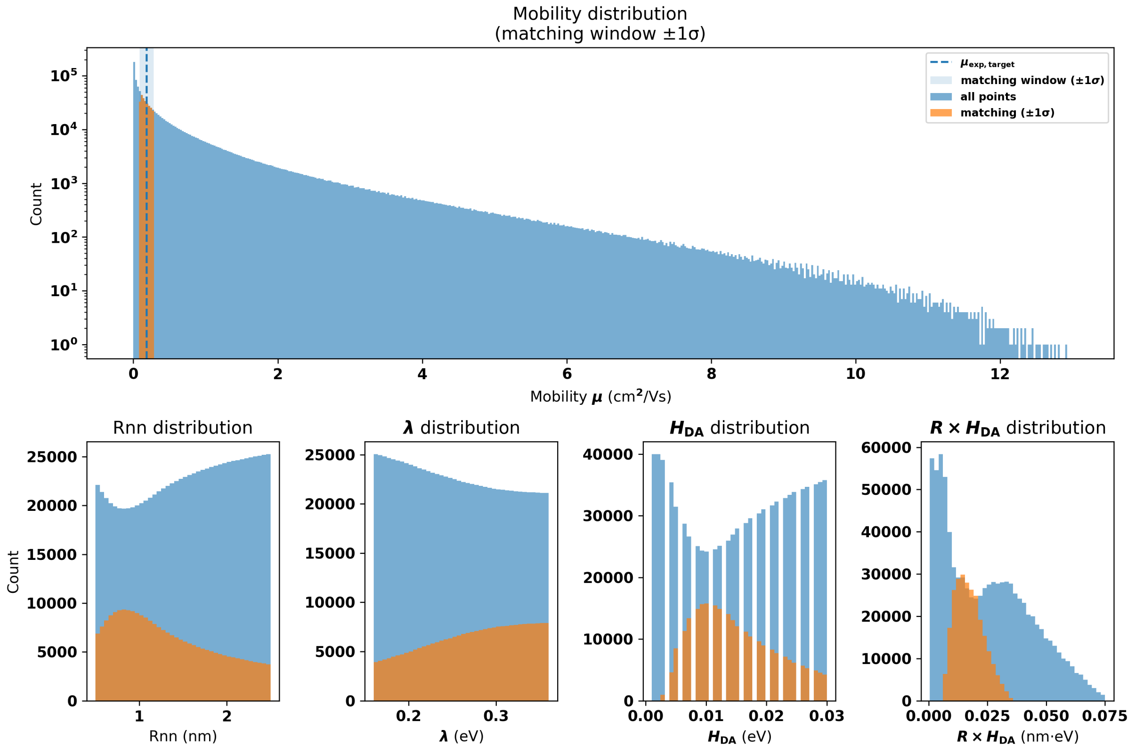

Figure S7: Panel (A) shows the distribution of calculated mobility values. In the orange is shown the mobilities which agree with the experimental average within one experimental standard deviation. Panels B, C, and D show the distribution of the Marcus parameter which produce mobilities that either are (orange) or are not (blue) consistent the experimental mobility within one standard deviation. Panels B-D show that a wide range of parameters across the allowed values could produce the experimental mobility when present in the right combination. Panel E shows the distribution of the combined inter-cofactor distance and the coupling. By grouping these two terms one can compare the distributions of quadratic terms in the mobility expression (coupling and distance) to the exponential terms (reorganization energy).

not produce experimentally consistent mobilities appear in Fig. S7B-E. These plots indicate that most Marcus parameter values that we considered, in combination with other in-range Marcus parameters, produced mobilities consistent with experiment. However, some of the lowest couplings sampled were not members of parameter sets that produced experimentally consistent mobility data (Fig. S7D). A coupling of at least $\sim 3\ meV$ is required to describe CB mobility data using a multi-step hopping model. Since both the squared coupling and inter-cofactor distance appear in the mobility, changing either value can be compensated by changing in the other one in proportion. The product of the coupling and distance must be between 0.00614 and $\sim 0.03574\ nm\ eV$ to agree with the experimental mobility (Fig. S7E). Values of $r_{nn} \cdot H_{DA} \geq 0.03574\ nm\ eV$ produce mobilities that are too high to be compatible with the measured experimental mobilities. That is, for $r_{nn} \geq 2.5\ nm$, the coupling must be $H_{DA} \leq 14.2\ meV$ for the calculated hopping mobility to be consistent with the experimental CB mobility data. On the lower end of the $r_{nn} \cdot H_{DA}$ range, which produce experimentally consistent mobilities, a coupling of $H_{DA} \leq 5\ meV$ would require $r_{nn} \geq 1.23\ nm$ for the calculated hopping mobility to be compatible with the experimentally reported average mobility within one standard deviation (as indicated in Figure S7E).

S7 The limit of junction conductivities lower than fiber conductivities

There is evidence that conductivities of the junctions are lower than, or at least different from, the conductivities of the fibers.[23] If the junctions are much less conductive than the fibers, the factor of 2/3 that corrects the 1D chain mobilities for the presence of conductive junctions can be neglected. Setting this correction factor to unity leads to acceptable Marcus parameters that replicate the experimental mobilities (see Fig. S8). The main differences between the case of uniform hopping throughout the CB (presented in the main text) and the case of low conductivity junctions (Fig. S8) are that the range of parameters for the low conductivity junctions necessitates

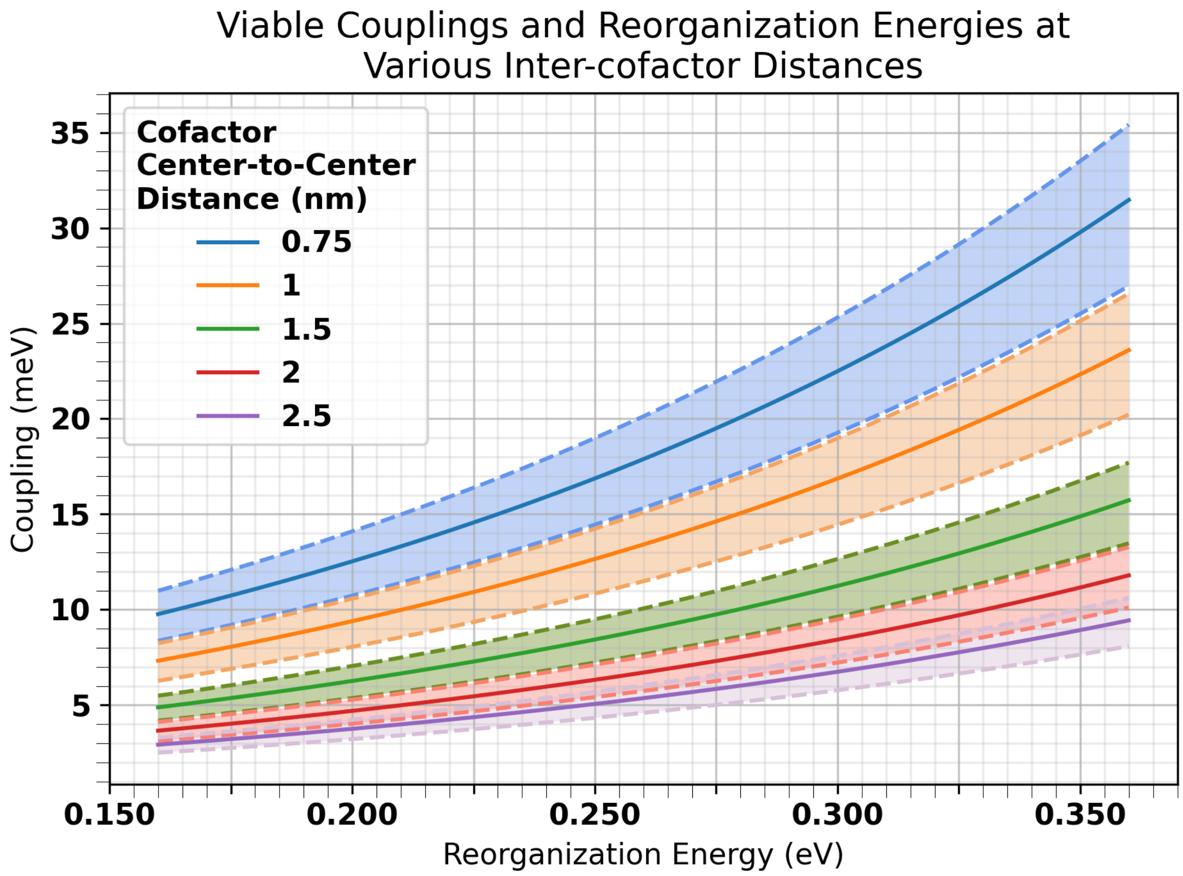

Figure S8: The hopping transfer parameters is shown for a CB in the limit where the junctions are much more resistive than the fibers, producing individual conduction pathways that behave as 1D transport chains.

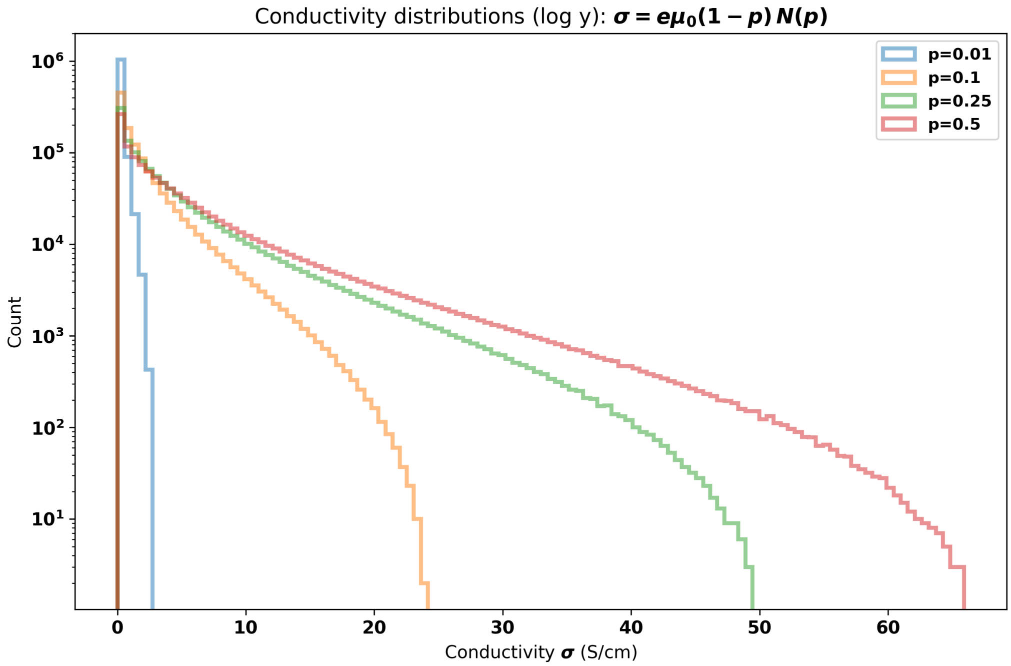

Figure S9: visThe distribution of conductivities produced for different values of the carrier density.

smaller couplings or smaller inter-cofactor distances than are found in the uniform conduction case.

S8 Conductivity and density of charge carriers

This section describes how the results of our study may be linked to CB conductivity measurements.

To compute a conductivity from a set of Marcus parameters, we use $\sigma = Ne\mu$, with the mobility expression in eqn (6) of the main text.

$$N = p/(\pi r_{nn}(d/2)^2) \tag{S8.1}$$

We model the charge carrier density in eqn (S8.1) by assuming that each conduction channel is a cylinder of diameter d ($d = 2nm$[7,11]), and each cofactor is a cylinder of the same diameter and of height r_{nn}.[11] p is the fraction of cofactors that is reduced. We also multiply eqn (6) of the main

text by a mean-field correction that accounts for higher particle densities (a multiplicative factor of $1 - p$).[24] Using this definition of the charge carrier density, we computed the conductivity for each Marcus parameter combination. This produces the conductivities shown in Fig. S9. Note that Fig. S9 is not expected to represent the experimental distribution of conductivities, as this would imply that CB exhibit all of the combinations of Marcus parameters tested. Rather, we assume that a narrow range of Marcus parameters is recruited in CB. As such, if a narrow range of Marcus parameters exists where the calculated mobility agrees with the experimental mobility, we consider it to be plausible that a Marcus mechanism could manifest in CB. We expect a narrow set of Marcus parameters to plausibly describe transport in CB, and to yield a narrow region of experimentally consistent conductivities. Since we find a narrow window of parameters that is consistent with experimental CB conductivities ($\sim 1-10\ S/cm$), we say that our model is consistent with the experimental conductivities. Interestingly, Fig. S9 shows that much larger conductivities ($\sim 30\ S/cm$) may be accessed in a Marcus-like hopping theory CB. While we believe our theory offers a satisfactory understanding of typical CB conductivities, a more complete theory would explain the full range of CB conductivities observed experimentally. Further experimental investigation into the mobilities of especially highly conductive CB could provide a better understanding of the source these high conductivity values ($> 50\ S/cm$). The fact that our modeling produces typical CB conductivities ($\sim 1-10\ S/cm$) at carrier densities of $p \leq 0.1$ supports the relevance of our low carrier density assumption.

S9 Relevance of transient localization theory to cable bacteria

Studies suggest that CB use a polaron-mediated charge transport mechanism, and that transient localization theory (TLT) may describe the transport.[12,25] While Pankratov et al.[12] proposed this mechanism with caution, it seems unlikely to describe CB transport based on the temperature-dependent mobilities and conductivities described in our analysis below. TLT was developed to

describe why high-purity single-crystal organic semiconductors may exhibit lower mobilities than expected from band transport theories.[26] TLT is intended to capture anti-Arrhenius transport mechanisms.[26] However, anti-Arrhenius charge transport is not consistent with the observed temperature dependence of mobilities found in CB.[5] As such, TLT seems unlikely to describe charge transport in CB. In this section of the SI, we explore the TLT model parameter requirements to produce an Arrhenius temperature dependence for the conductivity at temperatures up to 300K. We explain how TLT studies[25] indicate that such a high temperature Arrhenius temperature dependency in the conductivity requires electronic coupling between hopping sites in excess of 250 meV.

S9A Transient localization theory

Band theory suggests that defect-free organic crystals should be able to realize mobilities in excess of 10^3 cm^2/Vs.[26] In contrast, ultra-high-purity organic crystals are found to have mobilities of < 50 cm^2/Vs (with many high-purity organic crystals near 10 cm^2/Vs).[25,26] This indicates that the application of band theory to ultra-high-purity organic crystals may be flawed. TLT explains that the finding of mobilities lower than predicted from band theory for high-purity organic crystals may arise because phonons can assist with charge carrier localization.[25] The TLT description of the mobility posits that the wave function in a pure crystal is delocalized throughout the crystal at T = 0 K. As the crystal is heated, phonon modes will become populated, and TLT suggests that these phonon modes couple to electrons with sufficient strength to stabilize the formation of large polarons (in this context, larger than the unit cell).[27] While this polaron is delocalized over many molecules in the crystal (and may be large compared to expectations based on the Marcus small polaron theory), the extent of delocalization is much less than that of a fully delocalized wave function at T = 0 K. This localization, due to intrinsic dynamic thermal disorder, is what causes the reduction in mobility relative to the values predicted by band-theory.

S9B Applications of TLT to disordered hopping networks

In many implementations of TLT[25,27], a relaxation time approximation is used to calculate the mobility. This approach is similar to using the ergodic hypothesis (the time average of a quantity is equal to its ensemble average).[25] The relaxation time approximation in TLT prescribes that, instead of computing the average of the time-dependent localization length, we can instead compute the average the localization length at several static instances in time. This approximation is applicable when the disorder is of equal magnitude in the two treatments. In practice, the relaxation time approximation is realized by representing the system with the following Hamiltonian for a given instance of disorder.[25]

$$\hat{H}_k = \sum_i (\epsilon_i \hat{c}_i^+ \hat{c}_i + norm(0, \sigma_{static})) + \sum_{ij} (J_{ij}^0 + norm(0, \sigma_{dyn})) \tag{S9B.1}$$

Here, ϵ_i is the site energy, $\hat{c}_i^+$ ($\hat{c}_i$) is the creation operator (annihilation), J_{ij}^0 is the equilibrium - dynamic disorder absent - coupling, and $norm(0, \sigma_{dyn})$ is a random instance of dynamic disorder sampled form a normal distribution with mean zero and standard deviation σ_{dyn}. $norm(0, \sigma_{static})$ is a random instance of static disorder (the random disorder for different sets of site energies) sampled from a normal distribution of site energies with mean zero and standard deviation σ_{static}. In section S9B.1, we set all $J_{ij}^0 = J$ and all $\epsilon_i = \epsilon$.

Using the model Hamiltonian in eqn (S9B.1) , we can calculate the squared localization length (L_i) for a given random realization of the dynamic disorder:

$$L_k^2(\tau) = \frac{1}{Z} \sum_{n.m} \frac{2|\langle n|\hat{j}|m\rangle|^2 e^{-\beta E_n}}{(\hbar/\tau)^2 + (E_m - E_n)^2} \tag{S9B.2}$$

Here, Z is the partition function, $\hat{j}$ is the current operator, n and m are the eigenstates of the Hamiltonian, E_n and E_m are the corresponding Hamiltonian eigenvalues, and τ is the characteristic electronic fluctuation time scale (introduced from the relaxation time approximation discussed in ref.[25]). We compute the average squared localization length $\overline{L}^2(\tau) = \frac{1}{N} \sum_k^N L_k^2(\tau)$ and the mobility from eqn (S9B.2) :

$$\mu = \frac{e}{k_B T}\frac{\overline{L}^2(\tau)}{2\tau} \tag{S9B.3}$$

To evaluate the Hamiltonian in eqn (S9B.1) , we require that σ_{dyn} appropriately samples changes in the couplings driven by the phonons at a specific temperature. The variance in the coupling between sites i and j is calculated using:

$$\sigma_{dyn} = \sqrt{\frac{8\alpha^2(J^0)^2T}{2M\omega^2}} \tag{S9B.4}$$

Here, M is the molecular mass, α is the dimensionless electron-phonon coupling, J^0 is the average coupling, and ω is the phonon frequency. The remainder of the discussion below focuses on the analysis of Cuichi et al.[25] (see ref.[25] regarding choices of TLT parameters).

S9B.1 TLT mobility vs temperature

For TLT to be consistent with the temperature-dependent conductivity and mobility data for CB, TLT needs to produce Arrhenius temperature-dependent mobilities in the 50-300K temperature range. Most systems that are believed to exhibit TLT behavior have an anti-Arrhenius temperature-dependent mobility in this temperature range.[26] While this temperature dependence alone suggests that charge transport in CB poorly described by TLT. Ciuchi et al.[25] showed theoretically that systems with static disorder exhibit a transition from Arrhenius to anti-Arrhenius temperature dependency the coupling changes (see Fig. S10).

Ciuchi et al.[25] calculated the temperature dependent mobility as a function of disorder. They found that $\sigma_{static} = 0.5J^0$ has an Arrhenius to anti-Arrhenius temperature-dependent mobility transition at $T^* \sim 0.1J^0$. For the experimental CB temperature-dependent mobility/conductivity data to be explained by a TLT mechanism, and for the CB mobility to have the observed Arrhenius temperature-dependent conductivity/mobility, the transition to anti-Arrhenius temperature-dependent mobility must occur at a temperature above 300K (the maximum temperature of the experimental studies). By setting $T^* = 300K$ and solving for J^0, we find that a coupling of at

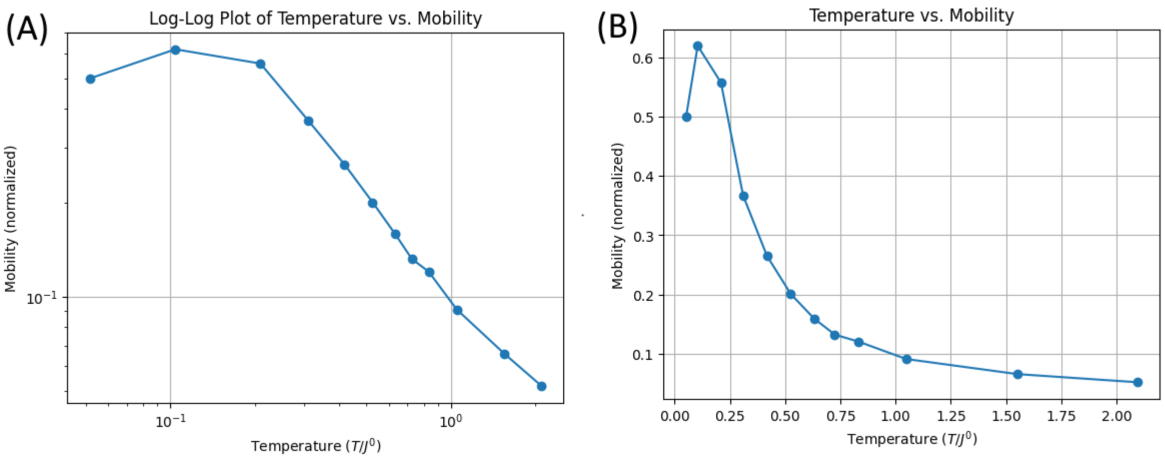

Figure S10: The figure shows the temperature-dependent TLT mobility data extracted from Ref.[25]. The mobility shown is for a system with $\sigma_{static} = 0.5J^0$ The TLT Arrhenius to anti-Arrhenius temperature-dependent mobility transition. For additional details describing the behavior and calculation of the mobility in TLT such as exact values of all parameters and mobility normalization procedure, see Ref.[25]. Panel (A) shows the temperature-dependent mobility in the same log-log plot as Ciuchi et al., and panel (B) shows the same data on a linear scale.

least 250 meV is required (with static disorder at least $\sigma_{static} = 0.5J^0$) to have $T^* \geq 300K$. As temperature-dependent conductivity data for CB does not generally exist above 300K, we cannot assess whether or not an Arrhenius to anti-Arrhenius temperature dependent transition consistent with TLT might occur above 300K. A 250 meV coupling between cofactors is much larger than is typical in biological systems (see main text), and the value is even large than is found in most covalently linked donor-acceptor structures (many synthetic systems have couplings in the 50-200 meV range).[28,29] Considering the high coupling required to produce Arrhenius temperature-dependent mobilities with a TLT-based charge transport mechanism in the 50-300K range, it seems that a TLT mechanism in CB is unlikely. However, we cannot rule out a TLT mechanism without knowledge of the electronic coupling interactions. In the main text, we showed that large couplings or biologically unlikely charge transport mechanisms are not required to explain the CB mobility data. That is, a multi-step (small polaron) hopping transport mechanism suffices to explain the reported data.

References

[1] A. Nitzan, *Chemical Dynamics in Condensed Phases: Relaxation, Transfer, and Reactions in Condensed Molecular Systems*, Oxford University Press, 2024.

[2] H. C. Berg, *Random Walks in Biology*, Princeton University Press, 1993.

[3] V. Stehr, J. Pfister, R. Fink, B. Engels and C. Deibel, *Phys. Rev. B*, 2011, **83**, 155208.

[4] K. Dill and S. Bromberg, *Molecular Driving Forces: Statistical Thermodynamics in Biology, Chemistry, Physics, and Nanoscience*, Garland Science, 2010.

[5] R. Bonné, J.-L. Hou, J. Hustings, K. Wouters, M. Meert, S. Hidalgo-Martinez, R. Cornelissen, F. Morini, S. Thijs, J. Vangronsveld *et al.*, *Sci. Rep.*, 2020, **10**, 19798.

[6] R. Cornelissen, A. Bøggild, R. Thiruvallur Eachambadi, R. I. Koning, A. Kremer, S. Hidalgo-Martinez, E.-M. Zetsche, L. R. Damgaard, R. Bonné, J. Drijkoningen *et al.*, *Front. Microbiol.*, 2018, **9**, 3044.

[7] L. Digel, M. L. Justesen, N. S. Madsen, N. Fransaert, K. Wouters, R. Bonné, L. E. Plum-Jensen, I. P. Marshall, P. B. Jensen, L. Nicolas-Asselineau *et al.*, *EMBO Rep.*, 2025, 1–19.

[8] F. J. Meysman, R. Cornelissen, S. Trashin, R. Bonné, S. H. Martinez, J. van der Veen, C. J. Blom, C. Karman, J.-L. Hou, R. T. Eachambadi *et al.*, *Nat. Commun.*, 2019, **10**, 4120.

[9] H. T. Boschker, P. L. Cook, L. Polerecky, R. T. Eachambadi, H. Lozano, S. Hidalgo-Martinez, D. Khalenkow, V. Spampinato, N. Claes, P. Kundu *et al.*, *Nat. Commun.*, 2021, **12**, 3996.

[10] T. Yang, M. S. Chavez, C. M. Niman, S. Xu and M. Y. El-Naggar, *eLife*, 2024, **12**, RP91097.

[11] J. R. van der Veen, S. Valianti, H. S. van der Zant, Y. M. Blanter and F. J. Meysman, *Phys. Chem. Chem. Phys.*, 2024.

[12] D. Pankratov, S. H. Martinez, C. Karman, A. Gerzhik, G. Gomila, S. Trashin, H. T. Boschker, J. S. Geelhoed, D. Mayer, K. De Wael *et al.*, *Bioelectrochemistry*, 2024, **157**, 108675.

[13] B. G. Lusk, S. Morgan, S. P. Mulvaney, B. Blue, S. W. LaGasse, C. D. Cress, J. T. Bjerg, W. K. Lee, B. J. Eddie and J. T. Robinson, *Proc. Natl. Acad. Sci. U.S.A.*, 2025, **122**, e2416008122.

[14] J. R. van der Veen, S. Hidalgo Martinez, A. Wieland, M. De Pellegrin, R. Verweij, Y. M. Blanter, H. S. van der Zant and F. J. Meysman, *ACS Nano*, 2024.

[15] J. Yang, S. Rahardja and P. Fränti, Proceedings of the international conference on Artif. Intell., information processing and cloud computing, 2019, pp. 1–6.

[16] R. Dawson, *J. Stat. Educ.*, 2011, **19**,.

[17] K. M. Sunderland, D. Beaton, J. Fraser, D. Kwan, P. M. McLaughlin, M. Montero-Odasso, A. J. Peltsch, F. Pieruccini-Faria, D. J. Sahlas, R. H. Swartz *et al.*, *BMC Med. Res. Methodol.*, 2019, **19**, 1–16.

[18] M. J. Crawley, *Statistics: an Introduction Using R*, John Wiley & Sons, 2014.

[19] G. Klein, *Proc. R. Soc. Lond. A.*, 1952, **211**, 431–443.

[20] F. Wang, Y. Gu, J. P. O'Brien, M. Y. Sophia, S. E. Yalcin, V. Srikanth, C. Shen, D. Vu, N. L. Ing, A. I. Hochbaum *et al.*, *Cell*, 2019, **177**, 361–369.

[21] M. J. Guberman-Pfeffer, *J. Phys. Chem. B*, 2023, **127**, 7148–7161.

[22] P. J. Dahl, S. M. Yi, Y. Gu, A. Acharya, C. Shipps, J. Neu, J. P. O'Brien, U. N. Morzan, S. Chaudhuri, M. J. Guberman-Pfeffer *et al.*, *Sci. Adv.*, 2022, **8**, eabm7193.

[23] R. Thiruvallur Eachambadi, R. Bonné, R. Cornelissen, S. Hidalgo-Martinez, J. Vangronsveld, F. J. Meysman, R. Valcke, B. Cleuren and J. V. Manca, *Adv. Biosyst.*, 2020, **4**, 2000006.

[24] M. A. Di Muro and M. Hoyuelos, *Phys. Rev. E*, 2022, **106**, 014139.

[25] S. Ciuchi and S. Fratini, *Phys. Rev. B*, 2012, **86**, 245201.

[26] S. Fratini, D. Mayou and S. Ciuchi, *Adv. Funct. Mater.*, 2016, **26**, 2292–2315.

[27] T. Nematiaram, S. Ciuchi, X. Xie, S. Fratini and A. Troisi, *J. Phys. Chem. C*, 2019, **123**, 6989–6997.

[28] L. Ueberricke, J. Schwarz, F. Ghalami, M. Matthiesen, F. Rominger, S. M. Elbert, J. Zaumseil, M. Elstner and M. Mastalerz, *Chem.–Eur. J.*, 2020, **26**, 12596–12605.

[29] H. Ishii, in *Charge Transport Simulations for Organic Semiconductors*, John Wiley Sons, Ltd, 2019, ch. 1, pp. 1–23.
